# Well-differentiated angiosarcoma of spleen: a teaching case mimicking hemagioma and cytogenetic analysis with array comparative genomic hybridization

**DOI:** 10.1186/s12957-015-0716-1

**Published:** 2015-10-13

**Authors:** Lichen Xu, Yimin Zhang, Hong Zhao, Qingxiao Chen, Weihang Ma, Lanjuan Li

**Affiliations:** State Key Laboratory for Diagnosis and Treatment of Infectious Diseases, Collaborative Innovation Center for Diagnosis and Treatment of Infectious Diseases, The First Affiliated Hospital, College of Medicine, Zhejiang University, 79 Qingchun Road, Hangzhou, Zhejiang 310003 China; Bone Marrow Transplantation Center, Department of Hematology, School of Medicine, Zhejiang University, Hangzhou, Zhejiang China; School of Medicine, Zhejiang University, Hangzhou, Zhejiang China

**Keywords:** Angiosarcoma, Well-differentiation, Splenectomy, Array comparative genomic hybridization, Copy number change

## Abstract

Primary splenic angiosarcoma is extremely rare but aggressive malignant vascular neoplasm. Here, we report a case of vascular tumor in spleen that was initially misinterpreted as hemangioma. Two years after splenectomy, the patient admitted again with aggravated abdomen pain and severe anemia. The magnetic resonance imaging (MRI) scan showed widely metastases. The ensuing biopsy for lesion both in liver and in bone marrow showed the similar pathological findings as that in spleen, which supported the final diagnosis of well-differentiated splenic angiosarcoma with extensive metastases. The patient was dead in 3 months after discharge without chemotherapy. The copy number changes for spleen lesion detected by array comparative genome hybridization showed copy number gain at 11q23.2, 11q24.3, 12q24.33, 13q34, copy number loss at 1q24.2-q31.3, 1q41-q42.2, 1 q42.3-q43, 2q36.3-q37.3, 2q37.7, 3q13.33-q26.2, 3q28 - q29, 9p11.2, 13q11, 15q11, homozygous copy loss at 8p11.22, 22q11.23. Less than 200 cases of splenic angiosarcoma have been published in literature of English. To the best of our knowledge, this is the first time analyzed cytogenetic alteration in a well-differentiated primary splenic angiosarcoma.

## Background

Primary splenic angiosarcoma (PSA) is a rare malignant neoplasm of endothelial cell, originating from vascular or lymphatic. PSA was first reported by Langhans in 1879. To date, no more than 200 cases of splenic angiosarcoma have been reported in literature [1]. The morphology features for this tumor are often uncharacteristic, ranging from well-formed anastomosing vessels to high-grade epithelioid or spindled cells without clear vasoformation [2]. Moreover, multiple appearances can be seen in the same case, making great challenge for diagnosis. However, no comprehensive research of cytogenetic changes to this subgroup of angiosarcoma has been published.

In this article, we describe a 38-year-old female patient of malignant vascular tumor that was initially interpreted as benign hemangioma in spleen with subtle cytological atypia and died 2 years later for multiple organ metastases. At the same time, we provided further array comparative genome hybridization (aCGH) analysis to make a preliminary exploration on the earlier identification of subgroup of angiosarcoma. To date, on cytogenetic analysis specific for primary splenic angiosarcoma has been reported.

## Case presentation

**Fig. 1:**
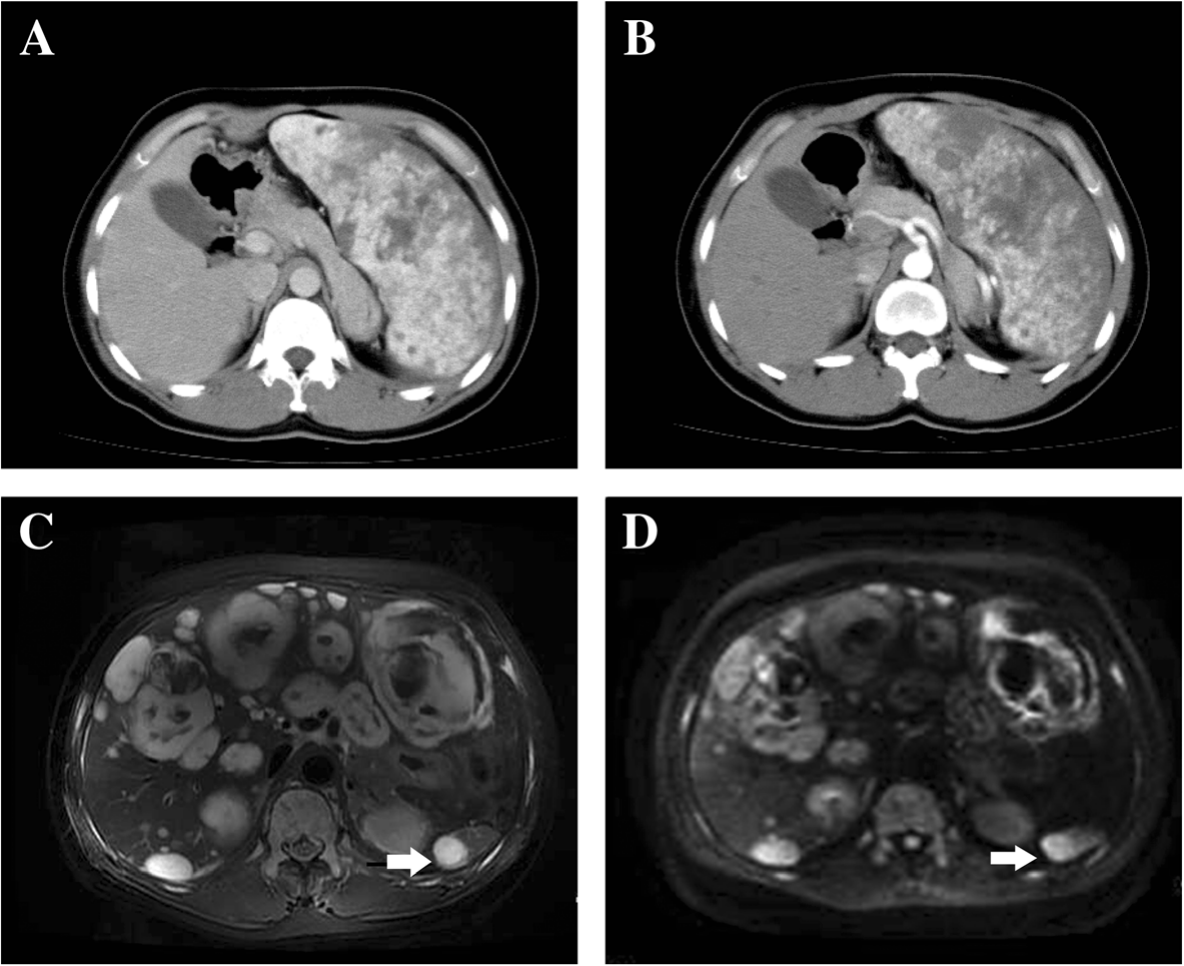
Abdomen CT scan in 2011 (**a, b**) demonstrated splenomegaly and multiple irregular low-density lesions. Liver, renal, adrenal, and pancreas are normal. The MRI in 2013 presented multiple cystic lesions in different sizes diffused in liver. The lesions displayed as inhomogeneous mixed signal, with high signal intensity on T2-weighted image (T2WI, **c**) and diffusion-weighted image (DWI, **d**). Some lesions that indicated eccentric or central low signal intensity were suspected intertumoral hemorrhage. Nodule lesion with abnormal sign as that in the liver was also found in remnant accessory spleen (*arrow*)

**Fig. 2:**
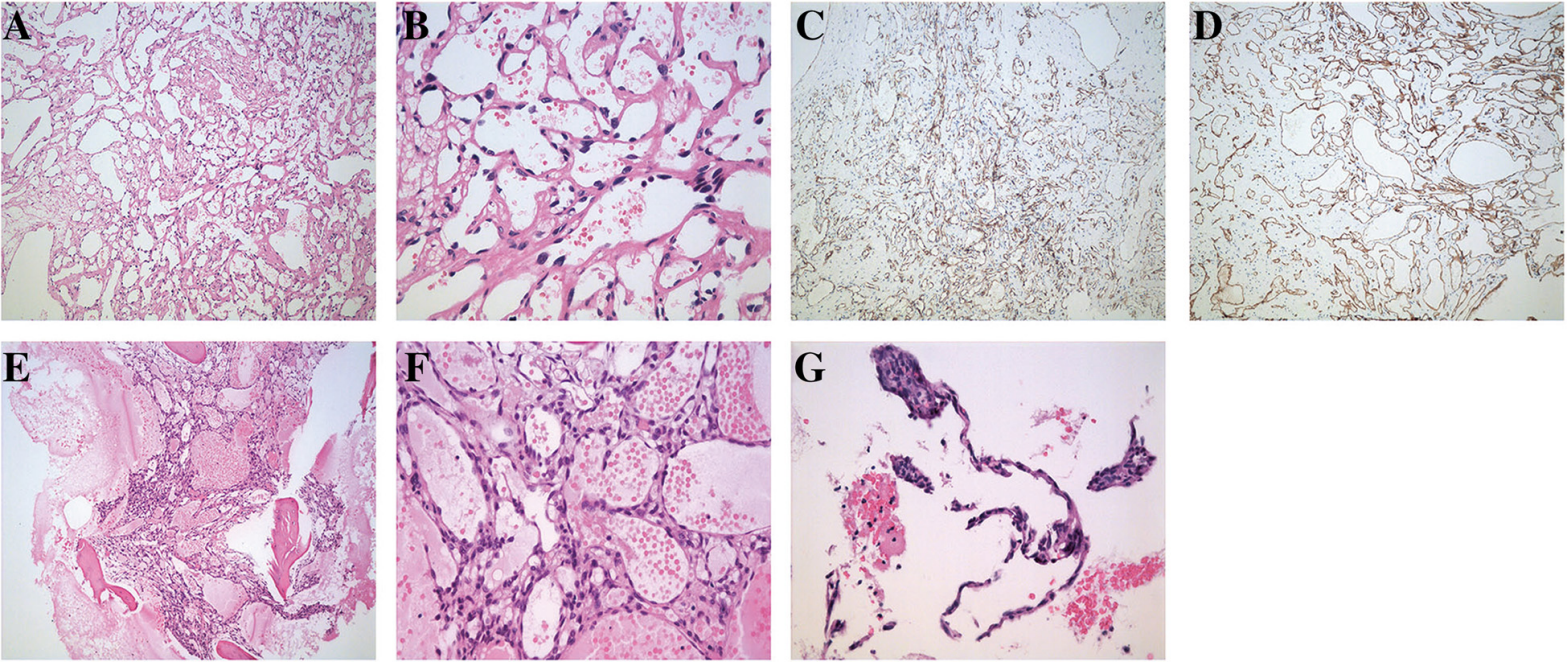
Tumor in spleen shows well-formed anastomosing vessel channels (**a**, HE 100×), proliferated endothelial cells with mild atypia (**b**, HE 400×), immunohistochemistry revealed positive staining of CD31 (**c**, 100×) and CD34 (**d**, 100×). Tumor cells from bone biopsy showed anastomosing vessel channels with proliferated endothelial cells without obvious malignant as that in spleen (**e**, HE 100×; **f**, HE 400×). Tumor cells from liver biopsy showed a handful of endothelial cells without obvious malignant (**g**, HE 100×)

A 38-year-old female patient was admitted to the hospital after suffering upper abdomen pain and fatigue for about 1 week in 2011. She has no history of smoking and alcohol or drug abuse. The patient denied immunodeficiency, history of malignancy and toxin exposure, and family history of genetic disorders. The abdomen computed tomography (CT) (Fig. 1) has been performed for further examination. The result showed splenomegaly with multifocal low-density lesions. Other organs including liver, pancreas, renal, and adrenal were normal. Considering possibility of splenic neoplasm, and for further clarification, splenectomy was carried out. Histopathology (Fig. 2) evaluation showed well-formed anastomosing vessel channels and proliferated endothelial cell with mild atypia but inconspicuously malignant. Immunohistochemistry (Fig. 2) revealed positive staining of CD31and CD34 but negative staining of factor VIII, CD68, p53. The pathology findings supported the diagnosis of hemangioma. Although no necrosis or hemorrhage was found in macroscopic and histological examination, in consideration of splenomegaly and anastomosing capillary-sized vessels, the well-differentiated angiosarcoma cannot be excluded. After splenectomy, the patient was quickly relieved from the pain and discharged without any postoperative complications. Then, the patient got lost to follow-up.

**Fig. 3:**
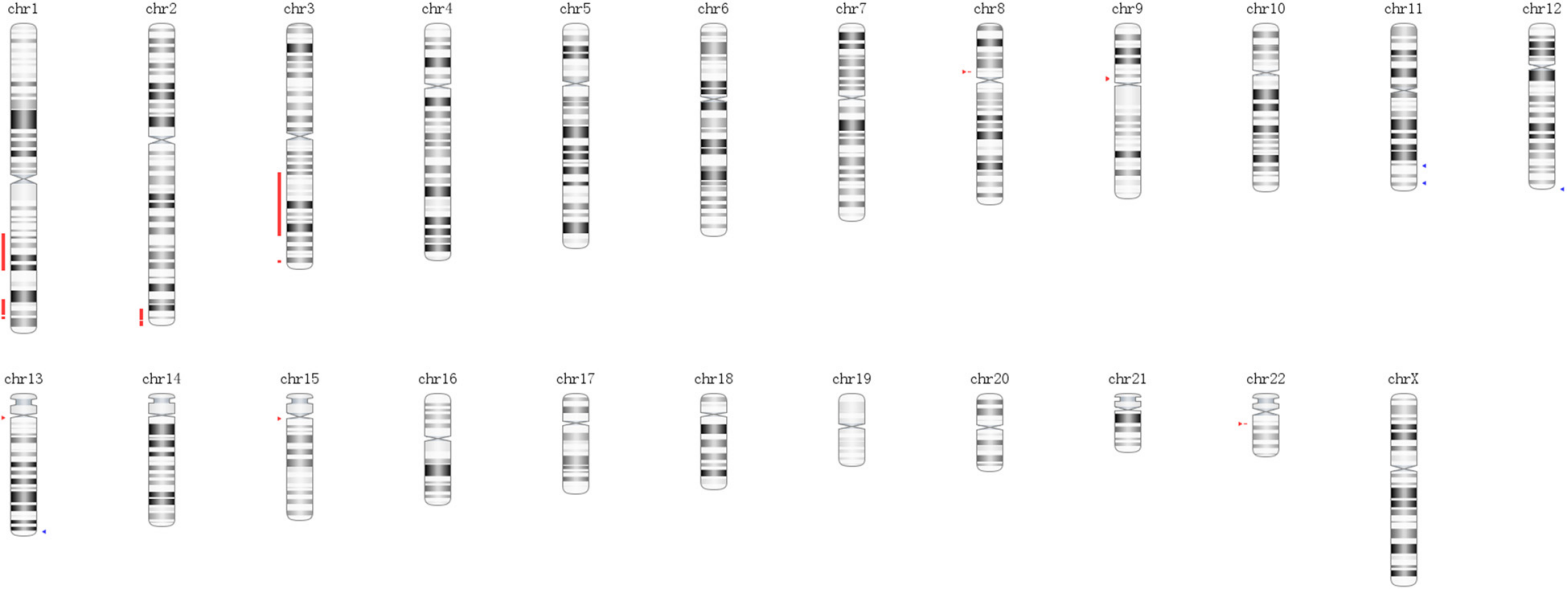
Summary of chromosomes copy number changes of splenic angiosarcoma detected by aCGH. The *blue color* on the right side represents copy number gains and *red color* on the left side represents copy number losses

Two years after splenectomy in December 2013, the patient presented with worsening upper abdomen pain, fatigue, and frequent dizziness for 3 months. On physical examination, she showed severe anemic appearance. There were no clinically palpable node and no papilledema. There was no evidence of bruising and easy bleeding. The initial lab tests were as follows: low hemoglobin (45 g/L, normal range 113–151), an increase of D-dimer, 78,700 ug/L (normal range 0–700ug/L), and a mild increase of aspartate aminotransferase (AST), 48 U/L (normal range 8–40U/L). Further examination of upper abdominal magnetic resonance imaging (MRI) was implemented. The result (Fig. 3) showed multiple nodes in the liver and accessory spleen with rich blood supply and signals of intratumoral hemorrhage. With these findings in MRI, fine-needle biopsy was obtained of the hepatic lesions. Due to further clarification of anemia, bone marrow trephine biopsy as well as smear for cytological examination were performed. The pathology evaluation (Fig. 2) on hepatic demonstrated a small amount of proliferated endothelial cells. The result of pathology findings of bone marrow biopsy (Fig. 2) showed anastomosing vascular channels, proliferated endothelial cells with few mitotic figures and mild nuclear atypia. The similarity of pathological findings in the liver, spleen, and bone marrow and clinical image presentation supported that the final diagnosis was well-differentiated angiosarcoma of the spleen with bone and liver metastasis. The patient was initially treated with analgesia and blood transfusion. Because few data can be acquired to guide chemotherapy and poor therapeutic effect, she gave up further chemotherapy after discussing with her family members. She was discharged in a few days after slight relief from pain and fatigue. Follow-up showed she passed away 3 months after discharge.

DNA was extracted from ten 20-μm thick ribbons of paraffin-embedded tumor of spleen from FFPE tissue blocks using DNeasy Blood &Tissue Kit (Cat#69506, QIAGEN, GmBH, Germany), following the manufacturer’s instructions. A total of 80 ng sample of DNA was sent for processing via OconScanTM FFPE Express 2.0 service as manufacturer’s instructions. The OncoScan assay contains more than 300,000 copy number and single nucleotide polymorphism (SNP) oligonucleotide probes with a median probe spacing of 4200 kb, with 541 somatic mutations for known cancer genes. The arrays were scanned by GeneChip® Scanner 3000 (Cat#00-00212, Affymetrix, Santa Clara, CA, USA) and Command Console Software 3.1 (Affymetrix, Santa Clara, CA, USA) with default settings. Raw data that passed quality control were further analyzed by Affymetrix® OconScan Analysis Suite (Affymetrix, Santa Clara, CA, USA).

Statistical analysis of the CGH data was performed by FASST2 Segmentation method. In order to adjust the sensitivity of the segmentation algorithm, we determined the significant threshold at 1.0E-8, specified 1000 kb being max contiguous probe spacing. The minimum number of probes per segment required to eliminate small CNVs was five; gains and losses were defined at ±3 × SD of all probes, and the threshold was adjusted at ±0.5 for both.

**Fig. 4:**
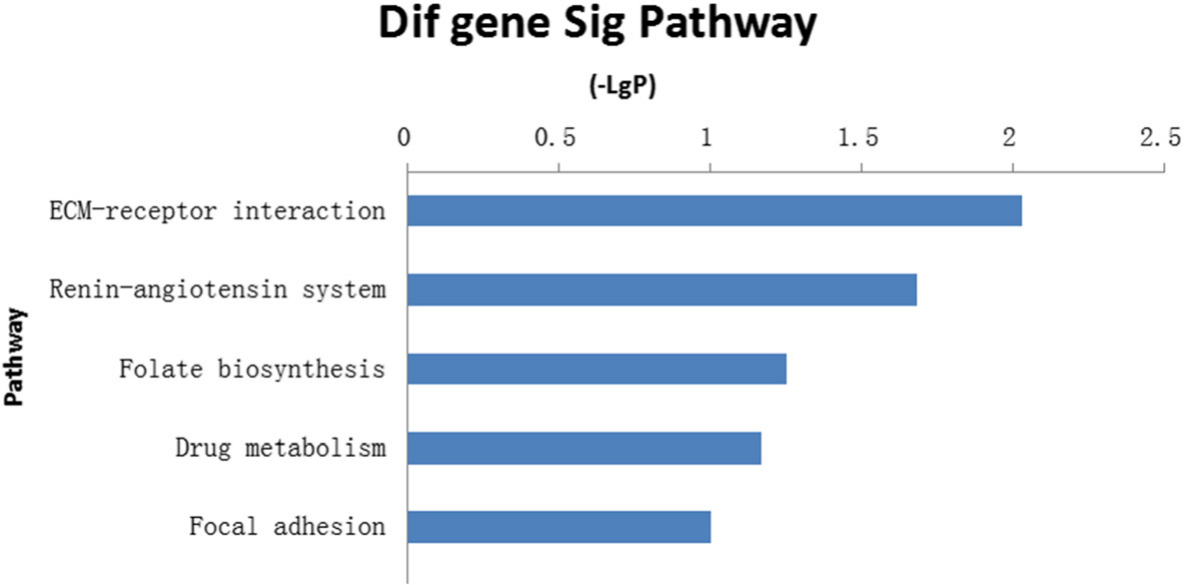
Gene ontology groups enriched by genes with copy number losses and gains. EASE score higher than 0.1 has been listed in the figure. The gene functional analysis revealed enrichments for otology terms related to diverse pathways, involved renin-angiotensin system and ECM-receptor interaction pathways signaling pathways

Copy number change of this angiosarcoma of spleen sample showed diverse DNA copy number alternations including copy number loss, copy number gain, and homozygous copy loss. The copy number changes were summarized in Fig. 3 and Table 1. The result (Fig. 4 and Table 1) revealed that copy number gain at 11q23.2, 11q24.3, 12q24.33, 13q34, copy number loss at 1q24.2-q31.3, 1q41-q42.2, 1 q42.3-q43, 2q36.3-q37.3, 2q37.7, 3q13.33-q26.2, 3q28-q29, 9p11.2, 13q11, 15q11, homozygous copy loss at 8p11.22, 22q11.23.

**Table 1:** Chromosomes copy number changes of splenic angiosarcoma

Chromosome	Location	Evens	Region length (bp)	Gene number
(a) Copy number (CN) loss or homozygous copy loss				
chr1	q24.2-q31.3	CN loss	30010137	238
chr1	q41- q42.2	CN loss	15946527	156
chr1	q42.3-q43	CN loss	19069070	21
chr2	q36.3-q37.3	CN loss	8571661	101
chr2	q37.7	CN loss	4012418	71
chr3	q13.33-q26.2	CN loss	50643792	428
chr3	q28-q29	CN loss	2176220	9
chr8	p11.22	Homozygous copy loss	197795	3
chr9	p11.2	CN loss	794282	1
chr13	q11	CN loss	289503	2
chr15	q11.1	CN loss	51427	0
chr22	q11.23	Homozygous copy loss	43891	4
(b) Copy number gain				
chr11	q23.2	CN gain	174597	1
chr11	q24.3	CN gain	427283	7
chr12	q24.33	CN gain	681673	20
chr13	q34	CN gain	520705	5

We found that a total of 1067 genes were affected by copy number changes according to the result of aCGH. In order to discuss the probably functions of altered genes, 397 out of 1067 genes with known biological functions, we used the Database for Annotation, Visualization and Integrated Discovery (DAVID) v6.7 (free online bioinformatics resources at http://david.abcc.ncifcrf.gov/) to clustered Gene Ontology (GO) group. The enriched GO groups (Fig. 4) were ranked according to statistical significance measured by EASE score (EASE score = −LgP), a modified Fish’s exact *p* value. The result illustrates that five relevant pathways have been affected by copy number changes as EASE score upper than 0.1. It is noteworthy that renin-angiotensin system and ECM-receptor interaction pathways exhibit statistically EASE score (*p* < 0.05).

## Discussion

Angiosarcoma, an extremely rare vascular malignant tumor, approximately composes 2 % of all soft tissue sarcoma. The incidence rate has risen over the past 30 years with unclear causes [3, 4]. PSA is the rare type of angiosarcoma. A tumor distribution analysis of 543 patients with angiosarcoma indicated PSA account for 2.5 % [3]. Clinical manifestation among PSA patient is variable and unspecific. Abdominal pain, weakness or fatigue, fever, chest pain, and/or weight loss, anemia, and thrombocytopenia were relatively predominant clinical presentation. Another, the sign related to metastasis like short of breath and gastrointestinal bleeding also can be the starting clinical manifestation when a patient was admitted [5–8]. Splenomegaly was commonly depicted in physical examination, and it can even be the only finding [6]. Some patients presented with celiac hemorrhage due to spontaneous splenic rupture, which occurred 13–32 % in the literature [6, 9]. Chemotherapeutic efficacy is still unclear but splenectomy as early as possible in people without metastasis may prolong period of survival [10].

Early metastasis was often taken place in high incidence with mostly involved in liver, bone marrow, lung, and lymph nodes. Aggressive progress and early metastasis always lead to dismal prognosis in patients with PAS. Unfortunately, histological findings cannot fully predict its malignant clinical behavior that even with brisk atypical histological changes may present metastatic lesions [11, 12]. Molecular tests have long been needed for diagnosis and classification. However, cytogenetic information on chromosomal abnormal on angiosarcomas are limited. In most cases, cytogenetic analysis indicated complex karyotypes, and the specific aberration characteristics for tumor development and behavior have far not been identified [13]. Gains in 8q and 20p and losses in 22q are the most frequent chromosomes imbalances among angiosarcomas [14]. But it should be kept in mind that the angiosarcomas in different anatomical sites or in different clinical settings might be based on distinguishing gene-expression profiling. A novel (1:14) (p21:q24) translocation has been reported in a primary bone angiosarcoma [15]. Cluster analysis of aCGH has identified two subgroup of primary bone angiosarcoma: a group with a complex genetic profile and a group with only few genetic aberrations [16]. Recent studies have shown that high-level amplification of MYC oncogene on chromosome 8q24.21 is specific for secondary angiosarcoma after radiation or lymphedema [17]. Co-amplification of FLT4 (encoding VEGFR3) on 5q35 was detected in 25 % of radiation-induced angiosarcoma [18]. Both FLT4 and MYC gene abnormalities can potentially be used as molecular diagnostic tool to distinguish from atypical vascular lesions (AVL) and secondary angiosarcoma [18, 19]. Loss of heterozygosity (LOH) of WT-1, RB1, and P53 locus at chromosome region 11p13, 13q14, and 17p13, respectively, was reported in angiosarcoma, supporting the role of those tumor suppressor genes in pathogenesis of angiosarcoma [20].

In the present case, we applied Affymetrix Onco-ScanTM FFPE Express molecular inversion probe microarray platform to analyze the chromosome abnormal. The result in this case was the same as reported elsewhere that angiosarcoma often displayed complex chromosome aberration [13]. The previous researches that benign vascular neoplasm often shows simple chromosome abnormality may support the malignant nature of the present case [21]. Notably, this observation raises possibility that even well-differential angiosarcoma may presents complex chromosome abnormality.

Aberration of 11q24.3 as seen in this case has reported in several malignancy. But the molecular mechanism in PAS remains unknown. Copy number gain in 11q24.3 may result in a constitutive high expression of ETS1 and FLI1 genes. EST1 was found up-regulated a number of angiogenic factors, such as vascular endothelial growth factor (VEGF), basic fibroblast growth factor (bFGF), angiotensin II, endothelin-1, tumor necrosis factor-α (TNF-α), or hydrogen peroxide. EST1 and FLI genes, ETS transcription factors, have been proved to play a significant role in malignancies [22, 23]. Conversely, inhibition of EST1 expression reduces the ability of endothelial cells to migrate, to proliferate, or to adopt an invasive behavior [23]. Strong expression of EST1 protein have been observed in angiosarcoma of skin, while it weakens in hemangioma [24]. FLI expression is a highly sensitive marker of a wide variety of common and unusual vascular tumors, but it cannot distinguish benign and malignant. A recent comprehensive study in diffuse large B cell lymphoma showed copy number gain in 11q24.3 and constructively up-regulates EST1 and FLI genes, which contributes to the pathogenesis of disease [25]. However, the roles of 11q24.3 amplification in PAS require further research both in molecular mechanism and in large sample investigation.

## Conclusions

In summary, primary splenic angiosarcoma remains to be a rare neoplasm with a high propensity for metastasis. It is still different to diagnose according to histopathology findings and immunohistochemistry profiles. But combined with radiological manifestation and microscopic examination, it may benefit to make final diagnosis. Cytogenetic analysis also gives some help to trace the malignant nature.

To the best of our knowledge, this is the first time that cytogenetic alteration was analyzed in a well-differentiated primary splenic angiosarcoma. However, the molecular features to characterize this subgroup of angiosarcoma need larger scale and more comprehensive study.

## Consent

The patient and the families were informed that data from the case would be submitted for publication and provided their consent accordingly.

## Abbreviations

MRI: magnetic resonance imaging
PSA: primary splenic angiosarcoma
aCGH: array comparative genome hybridization
CT: computed tomography
AST: aspartate aminotransferase
DAVID: database for annotation, visualization and integrated discovery
LOH: loss of heterozygosity
VEGF: vascular endothelial growth factor
bFGF: basic fibroblast growth factor
TNF-α: tumor necrosis factor-α
AVL: atypical vascular lesions
